# Rates of 1-year cognitive impairment in older adults who developed delirium due to a systemic infection

**DOI:** 10.1192/j.eurpsy.2021.379

**Published:** 2021-08-13

**Authors:** A.R. Silva, A.L. Cardoso, I. Baldeiras, I. Santana, J. Cerejeira

**Affiliations:** University Of Coimbra, Center for Neuroscience and Cell Biology, Coimbra, Portugal

**Keywords:** delirium, cognitive impairment, Hospitalization, dementia

## Abstract

**Introduction:**

Delirium affects a significant proportion of hospitalized older patients with acute infections. There is growing evidence that delirium accelerates the cognitive decline at long term.

**Objectives:**

We aimed to determine if delirium during hospitalization was independently associated with cognitive deterioration at one-year.

**Methods:**

From a total of 22 patients (12 C, 4 Dem, 2 D, and 4 DD) delirium (D and DD groups) was associated with a worse score in MOCA of 3-points (p<.02) and 2.5-points (p<.03), respectively, at one year, follow up. Dementia patients without delirium had a decrease of 2-point (p=.04) while cognitively healthy patients had a decrease in 1.08 points (p=.05) (Graph1). MOCA and NPI scores during hospitalization correlated significantly with cognitive decline in the four groups (r=.658, p<.01 and r=.439, p=.02, respectively.)

**Results:**

From a total of 22 patients (12 C, 4 Dem, 2 D and 4 DD) delirium (D and DD groups) was associated with a worse score in MOCA of 3-points (p<.02) and 2.5-points (p<.03), respectively, at one year follow up. Dementia patients without delirium had a of 2-point (p=.04) while cognitively healthy patients had a decrease in 1.08 points (p=.05) (Graph1). MOCA and NPI scores during hospitalization correlated significantly with cognitive decline in the four groups (r=.658, p<.01 and r=.439, p=.02, respectively.)

**Figure:**
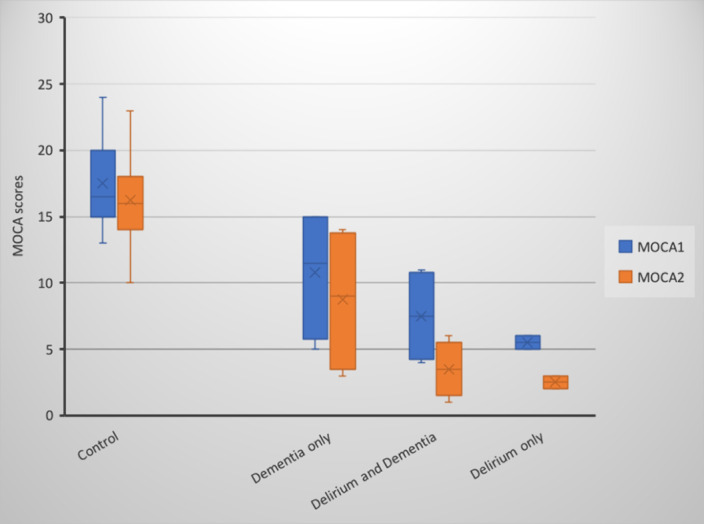

**Conclusions:**

Individuals developing delirium while recovering from infection have higher rates of cognitive decline after one year, but the cognitive decline is also present to a lower extent for individuals with infections that did not develop delirium.

**Disclosure:**

No significant relationships.

